# Time-series analysis with smoothed Convolutional Neural Network

**DOI:** 10.1186/s40537-022-00599-y

**Published:** 2022-04-26

**Authors:** Aji Prasetya Wibawa, Agung Bella Putra Utama, Hakkun Elmunsyah, Utomo Pujianto, Felix Andika Dwiyanto, Leonel Hernandez

**Affiliations:** 1 Electrical Engineering Department, Universitas Negeri Malang, Malang, 65145 Indonesia; 2 Faculty of Engineering, ITSA Institución Universitaria, Cra 45 No. 48-31, Barranquilla, Colombia

**Keywords:** CNN, Time-series, Exponential smoothing, Optimum smoothing factor

## Abstract

CNN originates from image processing and is not commonly known as a forecasting technique in time-series analysis which depends on the quality of input data. One of the methods to improve the quality is by smoothing the data. This study introduces a novel hybrid exponential smoothing using CNN called Smoothed-CNN (S-CNN). The method of combining tactics outperforms the majority of individual solutions in forecasting. The S-CNN was compared with the original CNN method and other forecasting methods such as Multilayer Perceptron (MLP) and Long Short-Term Memory (LSTM). The dataset is a year time-series of daily website visitors. Since there are no special rules for using the number of hidden layers, the Lucas number was used. The results show that S-CNN is better than MLP and LSTM, with the best MSE of 0.012147693 using 76 hidden layers at 80%:20% data composition.

## Introduction

Prediction estimates future events using a specific scientific approach [1] of analyzing time-series data patterns [2, 3]. One of the techniques is Convolutional Neural Network (CNN). CNN applies the basic concept of the Neural Network (NN) algorithm with more layers [4]. CNN is popular in computer vision and image processing for being efficient [5]. CNN uses a convolution layer that can handle spatial information available in images, while fully connected layers have a memory to store information in time-series data [6]. The only difference between computer vision problems and time-series ones is the input given to the model, image matrix for computer vision, and 1D array for time-series forecast [7]. The observation sequence can treat the raw input data as a 1D array that can be read and filtered by the CNN model. Thus, this principle can be implemented in time-serries analysis.

CNN deals with time-series problems effectively. Recent studies which applied CNN to time-series forecasting tasks, mainly involving financial data, show promising results. CNN estimates the stock market by extracting features and it can be used to collect data from various sources, including different markets models such as S and P 500, NASDAQ, DJI, NYSE, and RUSSEL [8]. The ability of convolutional layers for gold price volatilities may filter out the noise of the input data and extract more valuable features, which would be more beneficial for the final prediction model [9]. Many CNN models can solve various time-series data, such as univariate, multivariate, multi-step, and multivariate multi-step model [10].

CNN extracts image features from raw pixel data [11]. However, the raw data extraction is unnecessary in time-series analysis because of the numerical pattern. CNN may increase the accuracy up to 30% and train models twice faster than other algorithms such as RNN, GRU, and LSTM [12]. CNN weight division can reduce the number of parameters to increase the efficiency of model learning [13]. CNN is suitable for forecasting time-series because it offers dilated convolutions, in which filters can be used to compute dilations between cells. The size of the space between each cell allows the neural network to understand better the relationships between the different observations in the time-series [14].

Researchers have conducted various experiments to improve CNN performance. A novel approach which combined CNN with an autoregressive model outperformed both CNN and LSTM [15]. A specific architecture of CNN, WaveNet, outperformed LSTM and the other methods in forecasting financial time-series [16]. Livieris et al. [17] proposed a framework for enhancing deep learning by combining CNN-LSTM with simple exponential smoothing. The technique generated high-quality time-series data that considerably improves the forecasting performance of a deep learning model. Studies show that hybridizing CNN with other methods, creating a specific architecture, and smoothing the input data of CNN can increase the algorithm performance.

This study combines a simple exponential smoothing with CNN, Smoothed-CNN (S-CNN), to reduce forecasting errors. Instead of using a smoothing factor ($\alpha$), ranging from 0 to 1 in steps of 0.1 [17], this study promotes a novel optimum $\alpha$ as the main parameter of the simple exponential smoothing. We use CNN, Multilayer Perceptron (MLP), and Long Short-Term Memory (LSTM) with Lucas number hidden layers for the baseline and select the best method based on the performance analysis. Four different datasets are used to indicate the algorithm’s consistency.

The rest of this paper is organized as follows. “Smoothing algorithm for time-series” section describes details of the smoothing algorithm for time-series. “Experimental design” section presents the experimental design, focusing on the exponential dataset, data normalization, smoothing with optimum α, CNN with Lucas hidden layers, and performance testing. “Results” section presents the results and detailed experimental analysis, focusing on the evaluation of the proposed smoothing with optimum α. The section also summarizes the findings of this research by discussing the numerical experiments. Finally, “Conclusions” section summarizes the general findings of this study and discusses possible future research areas.

## Smoothing algorithm for time-series

Data smoothing can enhance the quality of data. Smoothing generates excellent results in small dataset forecasting by removing outliers from time-series data [18]. This method is easy to understand and can be effectively implemented in new research without referring to or taking parameters from other studies [19].

Smoothing procedures improve forecasting by averaging the past value of time-series data [20]. The algorithm assigns a weighting value to previous observations to predict future values [21], smooth the value of fluctuations in the data used, and eliminate noise [22]. Generally, there are four common types of data smoothing, which are simple exponential smoothing (SES)/exponential smoothing (ES), moving average (MA), and random walk (RW). In the case of a forecasting task, data smoothing can help researchers predict trends. Table 1 describes the types of data smoothing and the advantages and disadvantages.

**Table 1:** Types of data smoothing

Method	Advantages	Disadvantages
Exponential smoothing/simple exponential smoothing	• Ease of calculation • Flexibility • Good performance	• Not capable of managing trends well
Moving average	• Best used when there is slight or no seasonal variation	• Might not accurately reflect the most recent trends
Random walk	• Simple to use • Can easily handle flows around complicated boundaries	• Does not precisely conserve the mean position of the vorticity in free space • The computed solutions are noisy due to the statistical errors

In this study, we use exponential smoothing as a data smoothing method. Simple Exponential Smoothing (SES) [23], also known as Exponential Smoothing (ES) [18], was invented by Hyndman and is included in the R software’s libraries. Similar to other methods, ES works well for short-term forecasts that take seasonality into account, and the models chosen were evaluated only using MAPE. This method is currently used in a forecasting task due to its performance. Table 2 presents several related works which employ ES for forecasting. Exponential smoothing is a rule-of-thumb approach for smoothing time-series data using the exponential window function. Exponential functions are employed to apply exponentially decreasing weights over time. It is simple to learn and use for making a judgment based on the user's prior assumptions, such as seasonality [24].

**Table 2:** Related Works

Title	Year	Method	Resume
Convolutional Neural Network–Component Transformation (CNN–CT) for confirmed COVID-19 cases [25]	2021	CNN-CT (ARIMA and ES)	The combination of strategies outperformed most individual methods
A comparison between Seasonal Autoregressive Integrated Moving Average (SARIMA) and Exponential Smoothing (ES) based on Time Series Model for forecasting road accidents [26]	2021	SARIMA and ES	The ES model outperformed the SARIMA model of mean absolute error, and root mean square error, mean absolute percentage error, and normalized Bayesian information criteria
On short-term load forecasting using machine learning techniques and a Novel Parallel Deep LSTM-CNN approach [27]	2021	ARIMA, ES, Linear Regression, SVR, DNN, LSTM, LSTM-CNN, PLCNet	ARIMA and ES are two well-known time-series analysis approaches that need some parameter adjustment to work with these methods
A study on the prediction of power demand for electric vehicles using exponential smoothing techniques [28]	2021	ES and ARIMA	ES is 9% more accurate than ARIMA as a model of electric vehicle power-demand prediction models
Smoothing and stationarity enforcement framework for deep learning time-series forecasting [17]	2021	ES and CNN-LSTM	ES increases the deep learning forecasting performance
A hybrid method of exponential smoothing and recurrent neural networks for time series forecasting [18]	2020	ES-RNN	The winning hybrid method is used for data deseasonalization, normalization, and extrapolation
Forecasting time series with multiplicative trend exponential smoothing and LSTM: COVID-19 case study [29]	2020	MTES and LSTM	MTES outperformed LSTM in terms of RSME

## Experimental design

**Fig. 1:**
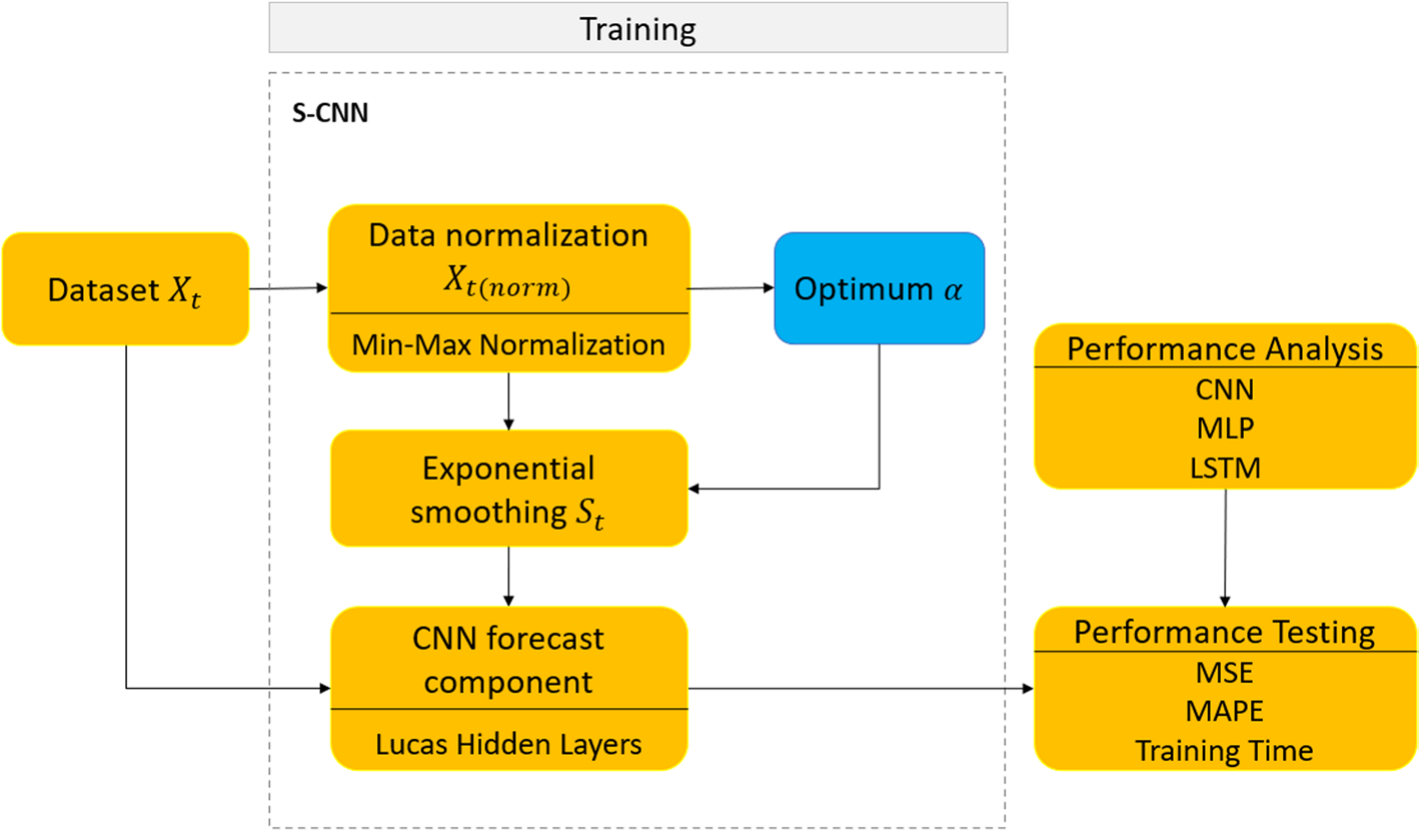
Experimental design of Smoothed-CNN (S-CNN) with optimum $\alpha$

In order to conduct a more systematic way of research, we designed the experiment as shown in Fig. 1. We generally compared the smoothed CNN with basic CNN using various datasets. We also used various scenarios and metrics to determine the best scenario. The details of Fig. 1 will be explained in the following subsections.

### Dataset

We used 4 different datasets in this study. Table 3 shows the characteristics of each dataset. Dataset 1 is the primary dataset, while the rest is used to test the proposed method’s consistency. All of the datasets were multivariate. However, we selected a single attribute (univariate) on each dataset due to the limitation of the study.

**Table 3:** Dataset Description

	Dataset 1	Dataset 2	Dataset 3	Dataset 4
Dataset Characteristics	Multivariate	Multivariate	Multivariate	Multivariate
Attribute Characteristics	Numeric	Numeric	Numeric	Numeric
Instances	365	365	365	365
Attributes	4 (Sessions, page views, visitors, new visitors)	4 (Page loads, unique visits, first time visits, returning visits)	2 (Sell, target)	32 cities in India
Missing data	No	No	No	No
Selected Attributes	Sessions	Unique visits	Sell	Delhi
Sources	http://journal2.um.ac.id/index.php/keds	https://www.kaggle.com/bobnau/daily-website-visitors	https://www.bi.go.id/en/statistik/informasi-kurs/transaksi-bi/Default.aspx	https://www.kaggle.com/twinkle0705/state-wise-power-consumption-in-india

The first time-series data in this study is from a journal website of Universitas Negeri Malang (Knowledge Engineering Data Science/KEDS). We retrieved the.csv from the Statcounter machine connected to the web. The dataset contains data within the period of January 2018 to 31 December 2018 [30]. In this study, the input and output of forecasting algorithms are the sessions attribute. Sessions (unique visitors) are the number of visitors from one IP in a certain period [31]. The number of unique visitors is an essential success indicator of an electronic journal. It measures the breadth of distribution that will accelerate the journal accreditation system [32]. The second dataset is similar to the first instead of the source and the range of the data. The third and fourth datasets are foreign exchange and electrical energy consumption.

**Fig. 2:**
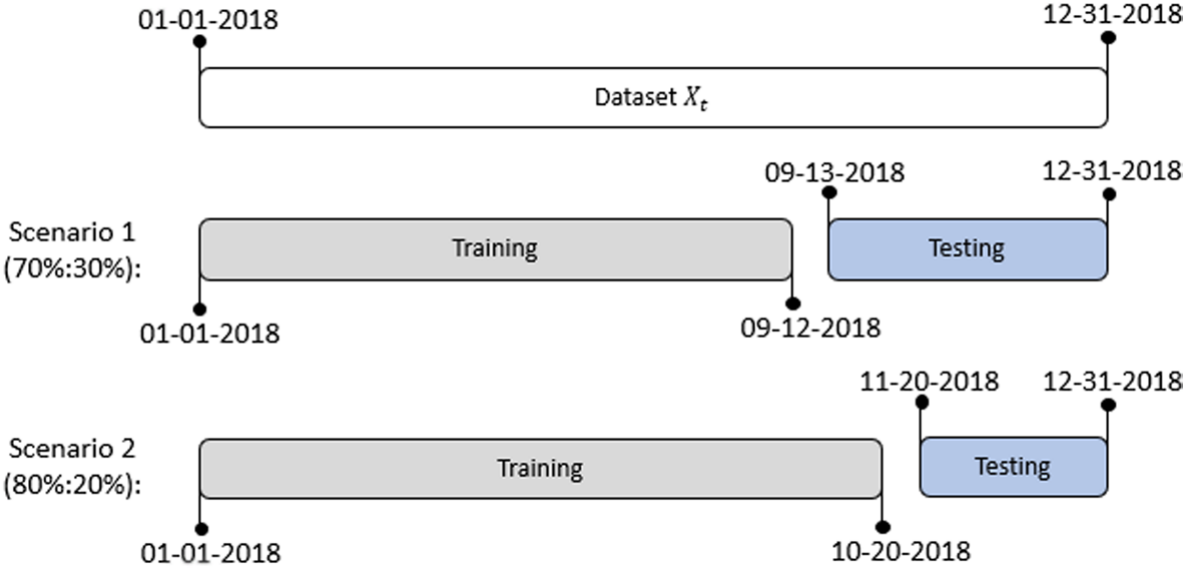
Training testing data composition

We used two scenarios to discover the influence of training data composition on forecasting performance. The first scenario used 70% (256 days) data training and 30% (109 days) data for testing. We used 80% (292 days) of the dataset as training in the second scenario while the rest was for testing (73 days). Figure 2 illustrates the scheme of training testing data composition.

### Data normalization

The natural behavior of most time-series is dynamic and nonlinear [33]. Data normalization is used to deal with this problem. Because the main objective of data normalization is to ensure the quality of the data before it is fed to any model, it substantially influences the performance of any model [34].

Data normalization is essential for CNN [35] because it can scale the attribute into a specific range required by the activation function. This study uses Min–Max normalization. The method assures that all features have the same scale, although it is inefficient in dealing with outliers. Equation (1) shows the Min–Max formula [36], resulting in normalized data with smaller intervals within 0–1.

1 $${X}_{t(norm)}=\frac{{X}_{t}-{X}_{ min}}{{X}_{ max}- {X}_{ min}}$$

${X}_{t(norm)}$ is the result of normalization, ${X}_{t}$ is the data to be normalized, while ${X}_{ min}$ and ${X}_{ max}$ stand for the minimum and maximum value of the entire data.

### Exponential smoothing with optimum $\boldsymbol{\alpha }$

In time-series forecasting, the raw data series is generally denoted as ${\{X}_{t}\}$, with starting time at $t=0$. Here t is a day index. The result of the exponential smoothing process is commonly written as ${S}_{t}$, which is considered as the potential future value of $X$. Equation (2) and (3) offer the single exponential smoothing [37] when $t=0$

$${S}_{0} ={ X}_{0}$$

2 $${S}_{t} ={\alpha X}_{t}+ \left(1-\alpha \right){ S}_{t-1 ,} t>0$$

3 $${S}_{t}={ S}_{t-1} +\alpha ({ X}_{t}-{ S}_{t-1})$$

The smoothed data ${S}_{t}$ is the result of smoothing the raw data ${\{X}_{t}\}$. The smoothing factor, $\alpha$ is a value that determines the level of smoothing. The range of $\alpha$ is between 0 and 1 (0 ≤ $\alpha$  ≤ 1). When $\alpha$ close to 1, the learning process is fast because it has a less smoothing effect. In contrast, values of $\alpha$ closer to 0 have a more significant smoothing effect and are less responsive to recent changes (slow learning).

**Fig. 3:**
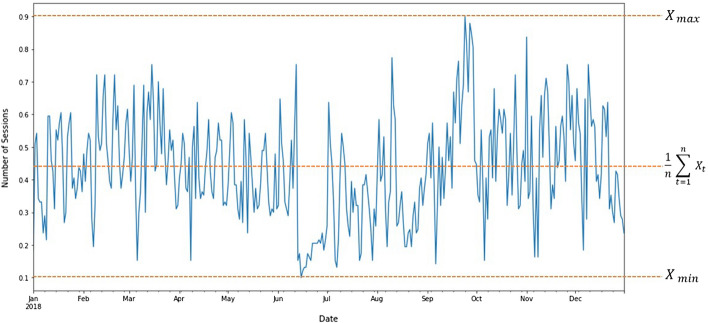
Time-series component of optimum $\alpha$

The value of $$\alpha$$ is not the same for every case. Therefore, we promote an optimum value of the smoothing factor based on the dataset characteristics. In this study, we use time-series data as in Fig. 3. Figure 3 shows the maximum ($${X}_{ max})$$, minimum ($${X}_{ min}$$), and average ($$\frac{1}{n} \sum_{t=1}^{n}{ X}_{t}$$) value of the series.

We have two considerations in order to define the optimum $\alpha$. The first is that the average value is less than the difference between ${X}_{ max}$ and ${X}_{ min}$. The second, the optimum $\alpha$ must be less than 1. Equation (4–6) shows the optimum $\alpha$ formula.

4 $$Optimum \alpha ={ \alpha }_{max}- \frac{\frac{1}{n} \sum_{t=1}^{n}{ X}_{t}}{{ X}_{ max}- { X}_{ min}}$$

5 $$Optimum \alpha =1-\frac{\frac{1}{n} \sum_{t=1}^{n}{ X}_{t}}{{ X}_{ max}- { X}_{ min}}$$

6 $$Optimum \alpha =\frac{\left({ X}_{ max}- { X}_{ min}\right)-\frac{1}{n}\sum_{t=1}^{n}{ X}_{t}}{{{X}_{ max}- X}_{ min}}$$

The substitution of Eq. (6) to (3) results in the following Eq. (7).

7 $${S}_{t}={ S}_{t-1} +\frac{\left({ X}_{ max}- { X}_{ min}\right)-\frac{1}{n}\sum_{t=1}^{n}{ X}_{t}}{{{X}_{ max}- X}_{ min}}({ X}_{t}-{ S}_{t-1})$$

We use the optimum smoothed result (${S}_{t}$) to improve the CNN algorithm performance.

### CNN with lucas hidden layers

CNN is the main algorithm of this research. CNN has the capacity to learn meaningful features automatically from high-dimensional data. The input layer used one feature since it is a univariate model. Flatten was used for input to get a fully connected layer. The fully connected layer contains dense for the number of hidden layers.

**Fig. 4:**
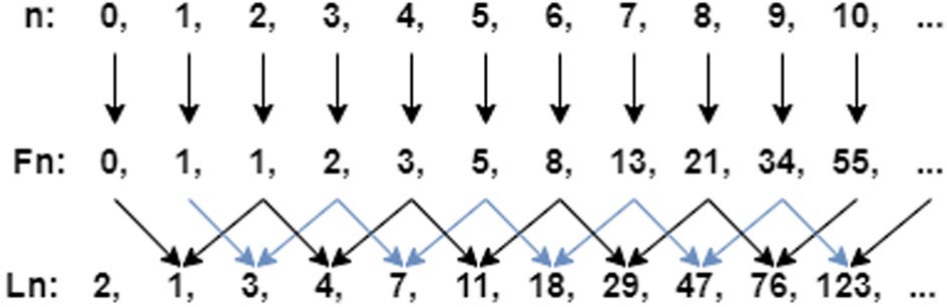
Sequence of Fibonacci and Lucas numbers

Instead of using a random number, we used the Lucas number to determine the hidden layer. The Lucas number (Ln) is recursive in the same way as the Fibonacci sequence (Fn), with each term equal to the sum of the two preceding terms, yet with different initial values. This sequence was selected since it provides a golden ratio number. The golden ratio emerges in nature, demonstrating that this enchanted number is ideal for determining the optimal solution to numerous covering problems such as arts, engineering, and financial forecasting [38]. To date, several computer science problems do not have an optimal algorithm. Due to the lack of a better solution in these circumstances, approaches based on random or semi-random solutions are frequently used. Therefore, using the Lucas number is expected to provide an optimal result, in this case, to determine a hidden layer [39]. Figure 4 presents the sequence of Fibonacci and Lucas numbers.

In this study, the Lucas number starts from three and ends with the last number before 100, which is 76. We limited the number of hidden layers to avoid the impact of time consumption and efficiency performance. Overall, we used 3, 4, 7, 11, 18, 29, 47, and 76 [40] for the numbers of hidden layers.

There are several lists of different parameters in CNN according to the layer. The CNN forecast component parameters can be seen in Table 4. The parameters selection is based on various research by Ref. [41–45]. A dropout was used during the weight optimization at all layers to avoid overfitting [46]. Dropout is a weight optimization strategy that randomly picks a percentage of neurons at each training period and leaves them out. The dropout value used was 0.2 [47].

**Table 4:** The list of CNN forecast component parameters

Category	Parameter	Value
Convolutional layer	Type of convolutional	Conv1D
	The number of convolutional layers	3
	The number of filters	128
	The filter size	2
	The activation function	ReLU
Pooling layer	Type of pooling	MaXPooling1D
	The number of pooling layer	1
	Size of the pooling window	2
Flattent layer	The number of flatten layer	1
Fully connected layer	The number of hidden layers	Lucas number
	The number of units or neuron	100
	The Activation function output	ReLU
	Loss function	MSE
	Type of optimizer	Adam
	The number of epochs	10,000
	The batch size	16

### Performance testing

All experiments in this study were performed using Python programming language from google collab executor using the Tensorflow and Keras libraries from google chrome browser. We used an Asus VivoBook X407UF Laptop with a 7th generation Intel Core i3 processor, 1 TB hard drive, and 12 GB DDR3 RAM.

We used the mean square error (MSE) and the mean absolute percentage error (MAPE) as error evaluation measures. The MSE was employed to identify anomalies or outliers in the planned projection system [48]. On the other hand, MAPE displayed mistakes that may signify correctness [49]. The formulae are as follows [49].

8 $$MSE = \sum\limits_{{t = 1}}^{n} {\frac{{(A_{t} - F_{t} )^{2} }}{n}}$$

9 $$MAPE = \sum\limits_{{t = 1}}^{n} {\frac{{|(A_{t} - F_{t} )|}}{{n.A_{t} }} \times 100}$$

${A}_{t}$ is the actual data value, ${F}_{t}$ is the forecast value, and $n$ is the number of instances. The better the forecasting outcomes, the less the MSE and MAPE value produced, and hence the better the approach utilized [50]. Based on the MSE and MAPE value computation results, the values show the best forecasting performance.

We also recorded the training time of every scenario. The information is used as additional performance indicators. We define the best algorithm as the method with the lowest time consumption.

## Results

Tables 5 and 6 present the comparison of CNN and S-CNN in all scenarios. We used $\alpha$ = 0.57 as the smoothing factor of the S-CNN. The hidden layers are various, starting from 3 to 76 of Lucas numbers. These layers were used for all scenarios, including the baseline: MLP and LSTM.

**Table 5:** MSE of CNN and S-CNN ( $\alpha$ = 0.57) in all scenarios

Number of Hidden Layers	Scenario 1		Scenario 2	
	CNN	S-CNN	CNN	S-CNN
3	0.039486471	0.036637854	0.015127435	0.015027847
4	0.038327266	0.035072528	0.022477422	0.020706612
7	0.026439827	0.022077946	0.019107487	0.017952291
11	0.025968207	0.025052203	0.029150361	0.023890096
18	0.024530865	0.020869428	0.017511018	0.017088791
29	0.026677929	0.025410001	0.017774397	0.016944807
47	0.026606092	0.026262877	0.013246628	0.013057781
76	0.026771594	0.020868076	0.013227105	0.012147693
Average	0.029351031	0.026531364	0.018452732	0.017101990

**Table 6:** Training time (s) of CNN and S-CNN ( $\alpha$ = 0.57) in all scenarios

Number of Hidden Layers	Scenario 1		Scenario 2	
	CNN	S-CNN	CNN	S-CNN
3	1401	1221	1901	1671
4	1451	1391	2021	1722
7	1551	1421	2121	1941
11	1650	1470	2380	2120
18	1810	1711	2630	2420
29	2120	1950	2840	2631
47	2601	2451	3241	2941
76	3521	3410	3641	3351
Average	2013	1878	2597	2350

Table 5 shows the MSE of CNN and S-CNN in all scenarios. Table 5 presents CNN results using the input data of Scenario 1 with a composition of 70% training and 30% testing data. From Tables 5 and 6, it can be seen that the average MSE value produced is 0.029351031, with an average processing time of 2013s. The highest MSE, 0.039486471, is achieved when the network has 3 hidden layers with a processing time of 1401 s. The lowest MSE 0.024530865 is generated when the hidden layer is 18, with a processing time of 1810s. The lowest MAPE is in the network with 3 hidden layers (10.38339615). Figure 4 shows the forecasting result of CNN within scenario 1.

**Fig. 5:**
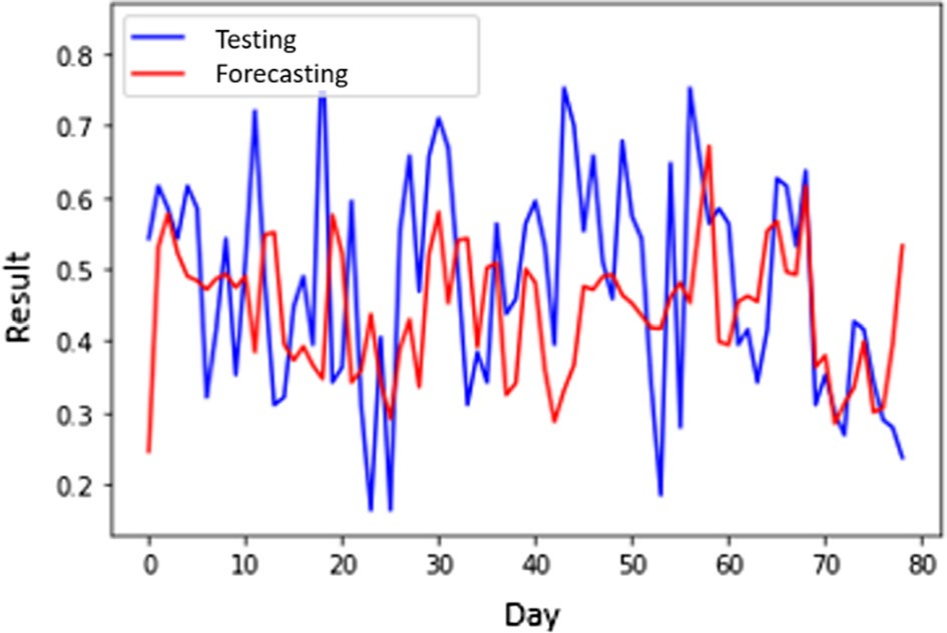
The forecasting results of CNN scenario 1

Tables 5 and 7 show that the number of hidden layers of the lowest MSE of smoothed CNN (S-CNN) is 76. The architecture has 0.020868076 MSE and 3410 s of processing time. The highest MSE, 0.036637854, is achieved when the network has 3 hidden layers with a processing time of 1221 s. This scenario generates the average MSE and processing time of 0.026531364 and 1878s, respectively. For scenario 1, S-CNN with 4 layers produces the best MAPE of 9.45147180. Figure 5 shows the forecasting result of S-CNN within scenario 1.

**Fig. 6:**
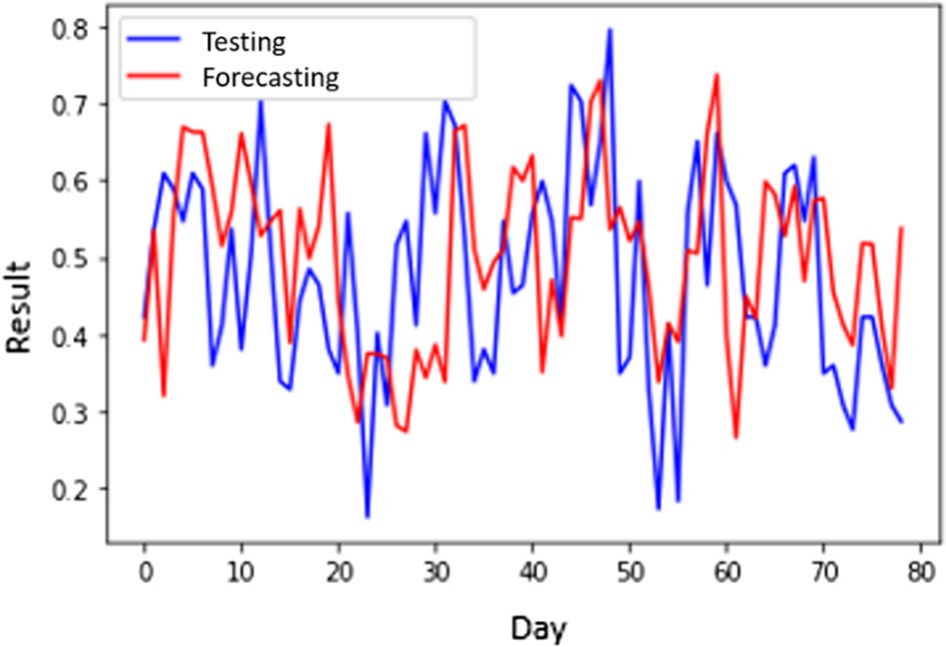
The forecasting results of S-CNN scenario 1

Figure 5 shows the best forecasting results because it has the lowest MSE. Despite the lowest MSE, from Fig. 5, we can see a fairly significant gap at the beginning and middle of the period. Meanwhile, when entering the end of the period, it can be seen in Fig. 5 that the forecasting results are similar to the original value. We can see that Fig. 6 shows a significant difference between the data and forecasting results at the middle and the end of the forecasting period. Nevertheless, the gap between testing data and the result in Fig. 5 is more significant than the gap between testing data and forecasting in Fig. 6. Thus, Fig. 6 is the best architecture due to its low MSE.

**Fig. 7:**
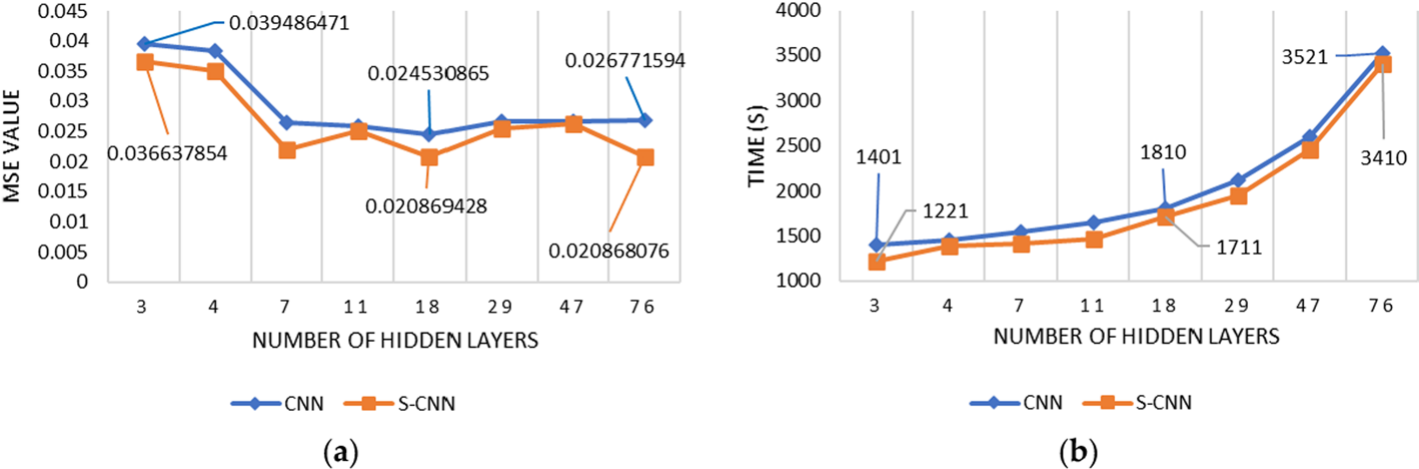
Comparison of CNN and S-CNN with scenario 1: **a** MSE; **b** Processing time

Figure 7 compares the CNN with the smoothed one. In general, S-CNN is better than the original CNN in terms of MSE. Figure 7a shows that the MSE of S-CNN is lower than CNN, except in the hidden layer 47, in which the MSE values of both are 0.026. The MSE values obtained by the two began to settle when they entered the hidden layer 7 to the last 76, with the average MSE value in that range being 0.026165752 for CNN and 0.023423422 for S-CNN. As Fig. 7b shows, the more hidden layers used, the longer the processing time required. When using the initial three hidden layers, the processing time is the same for both, 1142 s. Meanwhile, when using the last hidden layer, which is when using 76 hidden layers, the processing time required for S-CNN is 111 s faster than CNN. Again, S-CNN processing time is faster than CNN in every scenario.

**Fig. 8:**
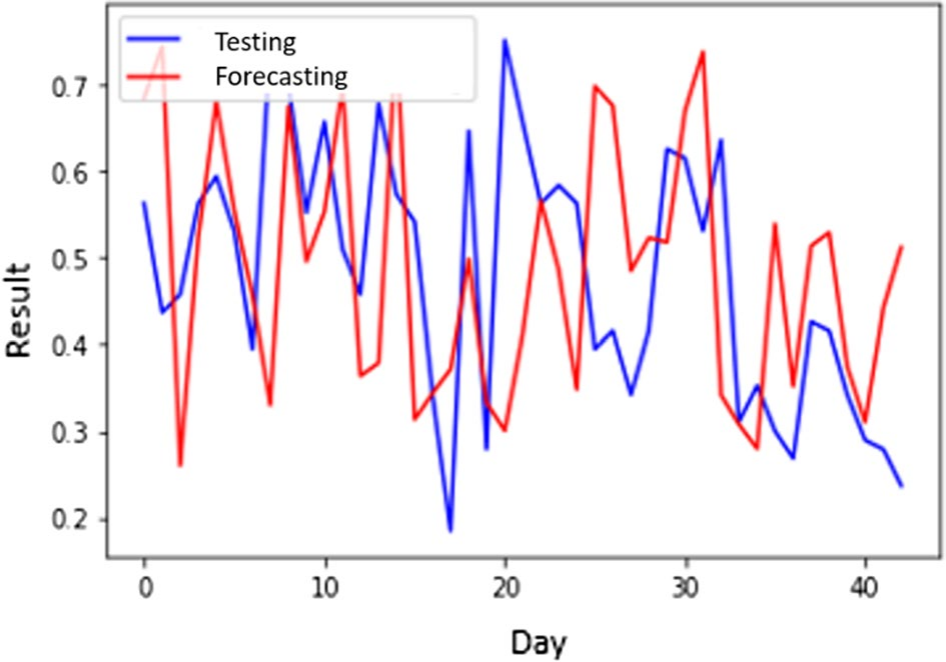
The forecasting results of CNN scenario 2

Table 5 show the CNN performance of Scenario 2 with 80% training and 20% testing data composition. The MSE results are 0.013227105 for the lowest and 0.018452732 for the average MSE. From Table 6, the average processing time is 2597 s, averaging the time between 1901 and 3641 s. The best structure for scenario 2 is using 7 hidden layers with MAPE = 9.29571771. Figure 8 presents the forecasting results of CNN within this scenario.

Figure 8 presents the best forecasting results which is the lowest MSE of Scenario 2. Despite the lowest MSE, Fig. 8 indicates a substantial disparity at the start and halfway. However, as Fig. 8 indicates, the forecasting results are approaching the initial value as the period ends.

**Fig. 9:**
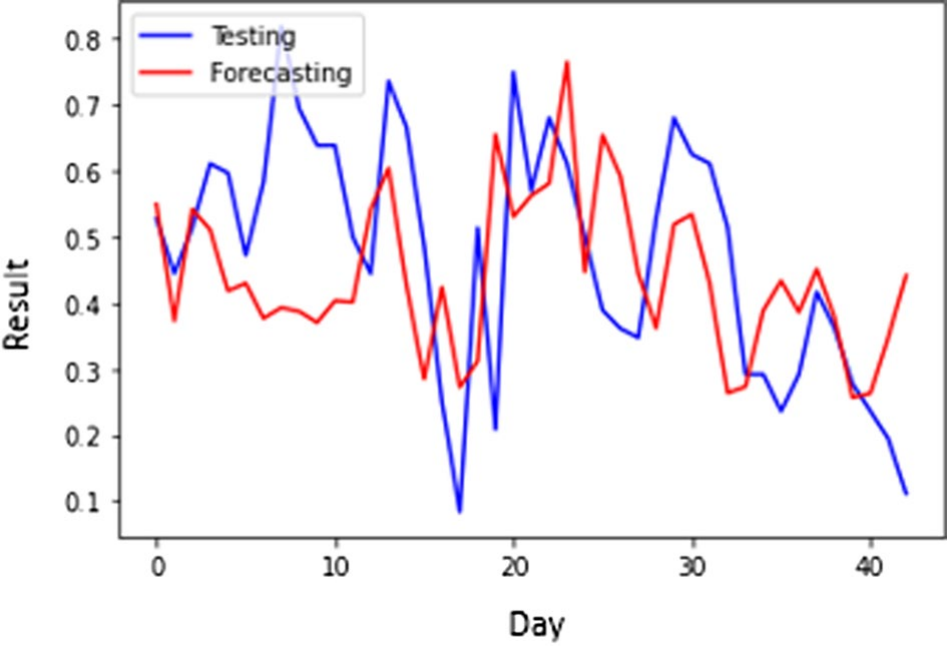
The forecasting results of S-CNN scenario 2

Table 5 presents the MSE and processing time of an S-CNN with the various hidden layers. The lowest MSE 0.012147693 happened when the hidden layer number was 76. Nevertheless, in Table 6, the computation is the longest among other architectures with 3351 s. The highest MSE was 0.023890096 due to 11 hidden layers. In Table 7, the lowest MAPE is 9.49165793 for the S-CNN with 29 layers. Figure 9 shows the forecasting results of the S-CNN.

**Table 7:** MAPE of CNN and S-CNN ( $\alpha$ = 0.57) in all scenarios

Number of Hidden Layers	Scenario 1		Scenario 2	
	CNN	S-CNN	CNN	S-CNN
3	10.38339615	10.15580773	10.32497287	9.86823878
4	10.63442349	9.45147180	10.23149490	10.26693096
7	10.55925727	9.48089667	9.29571771	10.58340192
11	10.44860005	10.49708843	10.37959695	10.50005768
18	10.86777925	10.29217958	10.22896290	10.13189130
29	10.48959136	10.39933443	10.74029326	9.49165793
47	10.60612321	9.936217308	10.73232293	9.50004619
76	10.66244721	10.57700276	10.75060725	9.70475220
Average	10.58145225	10.09874984	10.33549610	10.00587208

Figures 8 and 9 show a considerable change in the forecasted findings. Figure 8 has a greater MSE than Fig. 9, which means that the outlier occurrence of CNN is greater than S-CNN. In Fig. 8, the outliers occurs in almost all periods. On the other hand, the outliers occur in the early and late periods in Fig. 9. Therefore, it can be concluded that smoothing can improve performances by reducing the occurrence of outliers.

**Fig. 10:**
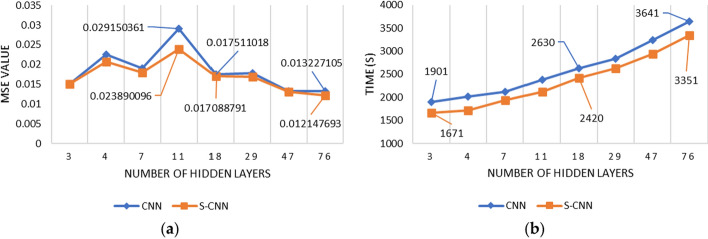
Comparison of CNN and S-CNN with scenario 2: **a** MSE; **b** Processing time

Figure 10 compares the CNN and S-CNN forecasting of Scenario 2 with the 80%:20% composition of the training and testing data. In general, Fig. 10a shows that the S-CNN has a lower MSE than its original version. It means that the CNN performance is less accurate than the S-CNN. On the other hand, the computation number of those methods is increasing in line with the rise of hidden layers numbers. In terms of processing time, smoothed CNN is faster than the original CNN in all scenarios, as seen in Fig. 10b.

We also compare our optimum α with α between 0.1 and 0.9 [17]. Table 8 shows the performance comparison using various values of smoothing factors. The results show that the optimum α has the lowest average MSE and MAPE. Therefore, our proposed optimum α outperformed other scenarios.

**Table 8:** Performance comparison using various values of smoothing factors

Alpha (α)	MSE		MAPE		Training Time (s)	
	Scenario 1	Scenario 2	Scenario 1	Scenario 2	Scenario 1	Scenario 2
0.1	0.041253364	0.037273699	10.53615943	10.38117275	1931	2565
0.2	0.040030418	0.036722839	10.52603677	10.40674120	1773	2459
0.3	0.037012976	0.032575541	10.47502294	10.42701104	1808	2354
0.4	0.033451774	0.028436491	10.27203577	10.21281549	1908	2470
0.5	0.028844447	0.019506374	10.26041789	10.04790929	1625	2622
0.6	0.028121199	0.019009872	10.32034706	10.23355049	1766	2466
0.7	0.027940839	0.019117107	10.20982302	10.12943395	1968	2285
0.8	0.028185234	0.020425496	10.32522286	10.22963096	1648	2566
0.9	0.028846332	0.018137853	10.35108365	10.13430657	1728	2444
0.57	0.026531364	0.01710199	10.09874984	10.00587208	1878	2350

Table 9 shows the significance of using Lucas numbers as hidden layers on MSE, MAPE, and training time. The significance is shown when the paired *t*-test 2-tailed P value < 0.05. The result shows that Lucas numbers have a significant impact on MSE and training time. The insignificance shown in the MAPE results means that the Lucas numbers hidden layers cannot significantly improve the accuracy.

**Table 9:** Paired *T*-test result based on lucas hidden layers

	MSE				MAPE				Training Time (s)			
	Scenario 1		Scenario 2		Scenario 1		Scenario 2		Scenario 1		Scenario 2	
	CNN	S-CNN	CNN	S-CNN	CNN	S-CNN	CNN	S-CNN	CNN	S-CNN	CNN	S-CNN
Lucas Hidden Layers	0.0312	0.0312	0.0311	0.0311	0.0709	0.0559	0.0608	0.0604	0.0001	0.0001	0.0001	0.0001

We also used paired t-test to indicate the significance of α on MSE, MAPE, and training time. Since the results in Table 10 are lower than 0.05, the use of α is significant to MSE, MAPE, and training time. In other words, using the smoothing factor is necessary to improve the forecasting performance.

**Table 10:** Paired *T*-test result based on alpha

	MSE		MAPE		Training Time (s)	
	Scenario 1	Scenario 2	Scenario 1	Scenario 2	Scenario 1	Scenario 2
Alpha (α)	0.0003	0.0003	0.0001	0.0001	0.0001	0.0001

The proposed CNN is compared with other time-series forecasting methods using the same dataset, preprocessing process, and general parameter settings. This study uses MLP and LSTM as the baseline. The general parameter setting for MLP and LSTM is the same as the CNN setting in Table 4. Table 11 shows the forecasting comparison of all approaches. In all scenarios, the CNN method has lower MSE and MAPE results than MLP or LSTM. Therefore, forecasting using smoothed CNN (S-CNN) has better performance than the original CNN.

**Table 11:** Forecasting comparison

Scenario	1 (70%:30%)			2 (80%:20%)		
Method	MSE	MAPE	Training Time (s)	MSE	MAPE	Training Time (s)
MLP	0.642934239	11.50568091	1340	0.635622626	10.94862916	1637
LSTM	1.701240209	11.39517313	2498	0.835000882	10.60269367	2927
CNN	0.029351031	10.58145225	2013	0.018452732	10.33549610	2597
S-CNN	0.026531364	10.09874984	1878	0.017101990	10.00587208	2350

We used three more datasets to test the consistency of the best algorithm, S-CNN. The best scenario is used to test the datasets: scenario 2, 76 hidden layers, and smoothing factor based on the statistical parameter of each dataset. The results of the evaluation using various types of datasets and different methods can be seen in Table 12. Table 12 presents the use of the S-CNN method on different datasets to find the best MSE and MAPE values. S-CNN outperformed the baseline in all datasets. The computation of S-CNN is more complex than other methods in Table 12. It is indicated that the time value is more significant than one in LSTM and MLP. Due to the smoothing process, S-CNN is slightly faster than its origin, CNN. Therefore, the results of the performance test are consistent in every dataset.

**Table 12:** Comparison with others Dataset

Method	MSE			MAPE			Training Time (s)		
	Dataset 2	Dataset 3	Dataset 4	Dataset 2	Dataset 3	Dataset 4	Dataset 2	Dataset 3	Dataset 4
MLP	1.846002201	5.650526501	0.910128122	10.98622048	9.98928011	9.92809521	3332	2720	3214
LSTM	1.844627951	5.597686482	0.577352403	10.98383963	9.98898726	9.93592591	3041	3263	3996
CNN	0.126788296	0.194362491	0.145794914	10.79435834	7.41526792	10.81731200	3911	3260	3420
S-CNN	0.100307581	0.170961120	0.101014294	10.60502933	7.30898141	10.68965315	3870	2660	3403

Overall, the proposed use of the optimum smoothing factor in CNN (S-CNN) may improve the forecasting performance of CNN by reducing the MSE and MAPE. The proposed smoothing factor is limited because it is suitable for seasonal time-series data. Second, the efficiency of the proposed algorithm for multivariate time-series analysis should be considered. Multivariate data has different ranges, units, and dependencies.

## Conclusions

This study aims to optimize the performance of CNN, a widely used algorithm for image processing, in time-series analysis. Based on the results of the analysis, it can be concluded that CNN with optimum smoothing factor performs better than other selected methods in time-series forecasting. The optimum alpha proposed in this study produces the best evaluation results. The use of Lucas numbers as hidden layers significantly raises the performance of the forecasting algorithm due to the generated golden ratio.

While the results have addressed the research objectives, this research still has limitations. The study is focused on implementing optimized exponential smoothing in fundamental deep learning methods. Therefore, the effect of implementing this method to more advanced deep learning algorithms (i.e., Resnet, hybrid CNN-LSTM) will be investigated in the future. Our next focus is a deeper analysis of different smoothing techniques for trend data and double or triple exponential smoothing implementation. The use of multivariate data will also be considered for further research.

## Data Availability

Attached in the submission.
